# Australia and New Zealand renal gene panel testing in routine clinical practice of 542 families

**DOI:** 10.1038/s41525-021-00184-x

**Published:** 2021-03-04

**Authors:** Hope A. Tanudisastro, Katherine Holman, Gladys Ho, Elizabeth Farnsworth, Katrina Fisk, Thet Gayagay, Emma Hackett, Gemma Jenkins, Rahul Krishnaraj, Tiffany Lai, Karen Wong, Chirag Patel, Amali Mallawaarachchi, Andrew J. Mallett, Bruce Bennetts, Stephen I. Alexander, Hugh J. McCarthy

**Affiliations:** 1 Faculty of Science and Faculty of Medicine and Health, The University of Sydney, Sydney, NSW Australia; 2 Department of Molecular Genetics, The Children’s Hospital at Westmead, Westmead, NSW Australia; 3 KidGen Collaborative, Australian Genomics Health Alliance, Parkville, VIC Australia; 4 Discipline of Genetic Medicine and Discipline of Child & Adolescent Health, Faculty of Medicine and Health, The University of Sydney, Sydney, NSW Australia; 5 Genetic Health Queensland, Royal Brisbane and Women’s Hospital, Herston, QLD Australia; 6 Department of Medical Genomics, Royal Prince Alfred Hospital, Sydney, NSW Australia; 7 Garvan Institute of Medical Research, Sydney, NSW Australia; 8 Department of Renal Medicine, Royal Brisbane and Women’s Hospital, Herston, QLD Australia; 9 Institute for Molecular Bioscience and Faculty of Medicine, The University of Queensland, Brisbane, QLD Australia; 10 Centre for Kidney Research, Kids Research Institute and the Department of Nephrology, The Children’s Hospital at Westmead, Westmead, NSW Australia

**Keywords:** Genetic testing, Kidney diseases, Disease genetics

## Abstract

Genetic testing in nephrology clinical practice has moved rapidly from a rare specialized test to routine practice both in pediatric and adult nephrology. However, clear information pertaining to the likely outcome of testing is still missing. Here we describe the experience of the accredited Australia and New Zealand Renal Gene Panels clinical service, reporting on sequencing for 552 individuals from 542 families with suspected kidney disease in Australia and New Zealand. An increasing number of referrals have been processed since service inception with an overall diagnostic rate of 35%. The likelihood of identifying a causative variant varies according to both age at referral and gene panel. Although results from high throughput genetic testing have been primarily for diagnostic purposes, they will increasingly play an important role in directing treatment, genetic counseling, and family planning.

## Introduction

Kidney disease can present in a number of ways in children and adults, either as isolated cases or with a family history^1^. Where the history is suggestive, detailed analysis of the kidney phenotype with particular consideration of extra-renal manifestations can implicate a genetic etiology. Genetic testing offers an important additional facet to the routine diagnostic work up. Several recent international reports of testing in large cohorts of non-specific chronic kidney disease (CKD) and more directed testing of children with kidney disease have shown diagnostic rates of between 9 and 42%^2–4^, approximately in line with our previous report on gene panel testing in 135 Australian and New Zealand families with a diagnostic rate of 43%^5^.

Many genetic kidney diseases demonstrate significant genetic heterogeneity and the introduction of massively parallel sequencing into research genomics has allowed for the delineation of many novel gene associations and causative pathogenic variants^6–17^. However, as data sharing tools such as ClinVar^18^ and Online Mendelian Inheritance in Man (OMIM)^19^ as well as multidisciplinary diagnostic teams identify an increasing number of associative variants and improve the assessment of clinical presentations, there is a growing need for these advances in research to be translated into effective and reliable diagnostic tools in the clinical setting.

Large cohort studies on the utility of exome sequencing in kidney disease diagnosis have been performed in cohorts of 3315 unselected American patients with chronic and end stage kidney disease^2^, 1001 Chinese children with kidney disease^3^, and 138 Irish adults with CKD^4^. As genetic sequencing becomes more accessible as a routine tool in the diagnostic work up of a patient or family with kidney disease, quantifying the use by clinicians provides an understanding of the need for future training and education in genomic literacy among nephrologists, both adult and pediatric based. Furthermore, publication of results of testing in an unselected regional cohort provides a reference for referring health professionals to gauge the likelihood of identifying disease-causing variants in each clinical scenario.

The Australia and New Zealand Renal Gene Panels (ANZRGP) service was established in 2013 at the Children’s Hospital at Westmead (Sydney, NSW, Australia). Accredited by the National Association of Testing Authorities and with the support of Australian Genomics and its kidney flagship KidGen, ANZRGP employs targeted exome sequencing for multigene panels associated with more than 22 kidney disease categories and over 230 different genes (Supplementary Table 1). Gene panel lists are regularly updated and identified variants are classified based on the 2015 American College of Medical Genetics (ACMG) guidelines^20^ by a multidisciplinary team (Methods). In this study, we evaluate the use of massively parallel sequencing in kidney disease by health professionals across Australia and New Zealand and quantify the frequency of disease-causing variant identification. We extend the analysis of the diagnostic utility of kidney gene panels from our initial report of 135 families in 2017^5^ to now include over 500 patients, the largest cohort of Australian and New Zealand kidney patients reported to date.

## Results

### Demographic data

**Fig. 1:**
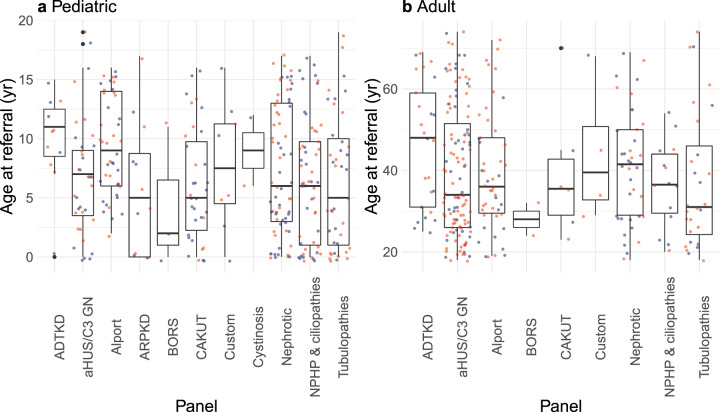
Ages at referral by panel. **a** Distribution of ages at referral in the pediatric cohort (*n* = 281 probands). **b** Distribution of ages at referral in the adult cohort (*n* = 271 probands). Each data point represents a proband (blue = male; red = female) referred for the relevant panel. Filled black points indicate outliers (points beyond 1.5 times the interquartile range above the upper hinge [75th percentile] or below the lower hinge [25th percentile]). Abbreviated panels are as follows: ADTKD autosomal dominant tubulointerstitial kidney disease, aHUS/C3 GN atypical hemolytic uremic syndrome-C3 glomerulonephritis, Alport Alport syndrome, ARPKD autosomal recessive polycystic kidney disease, BORS branchio-oto renal syndrome, CAKUT congenital anomalies of the kidney and urinary tract, Nephrotic nephrotic syndrome, NPHP & ciliopathies nephronophthisis & related ciliopathies.

**Fig. 2:**
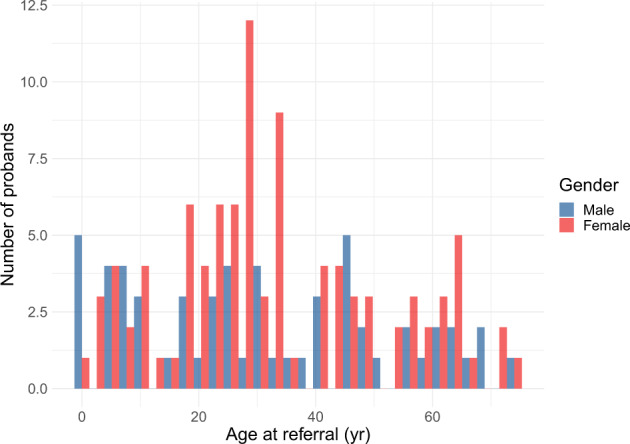
Age and gender distribution of 154 probands referred for atypical hemolytic uremic syndrome-C3 glomerulonephritis (aHUS/C3 GN) panel testing. Referrals were processed between December 2013 and October 2019.

**Fig. 3:**
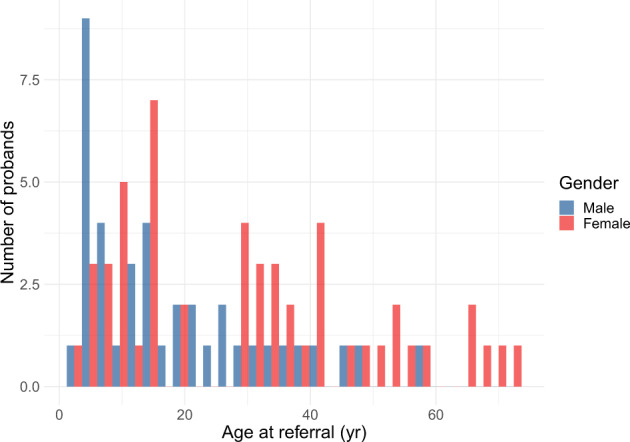
Age and gender distribution of 90 probands referred for Alport syndrome panel testing. Referrals were processed between December 2013 and October 2019.

552 individuals (271 adult and 281 pediatric patients) from 542 unrelated families from all Australian states and territories and New Zealand were referred. All individuals had consented for clinical genetic testing between December 2013 and October 2019. The overall median age at time of test request was 17 years (interquartile range [IQR], 30 years); the median pediatric age was 6 years (IQR, 8 years) and that of adults was 36 years (IQR, 22 years) (Fig. 1, Table 1). The overall sex distribution was 54% females and 46% males; the pediatric cohort was made up of 48% females and 52% males while the adult cohort was made up of 61% females and 39% males (Table 1). Demographic characteristics varied by panel (Table 1). For example, 173% more females than males between 20 and 30 years old were referred for atypical hemolytic uremic syndrome-C3 glomerulonephritis (aHUS/C3 GN) panel testing, possibly reflecting pregnancy-associated aHUS or thrombotic microangiopathy (TMA) (Fig. 2)^21^. In addition, the distribution of ages at referral was generally higher for females than males undergoing testing for Alport syndrome (Fig. 3). Patients were mainly referred by pediatric nephrologists (39%), adult nephrologists (33%), and clinical geneticists (23%).

**Table 1:** Summary of demographic and diagnostic results of the 542 families in Australia and New Zealand stratified by panel.

Panel	Number of patients (families)	%F	Median pediatric age at referral (IQR) (year)	Pediatric diagnostic rate (%)	Median adult age at referral (IQR) (year)	Adult diagnostic rate (%)	Overall diagnostic rate (%)
ADTKD	36 (35)	53	11 (4)	0	48 (28)	24	17
aHUS/C3 GN	154 (152)	62	7 (5.5)	23	34 (25.5)	17	18
Alport	90 (86)	57	9 (8)	64	36 (18.5)	67	65
ARPKD	12 (12)	50	5 (8.75)	25	−	−	25
BORS	5 (5)	60	2 (5.5)	33	28 (4)	0	20
CAKUT	40 (39)	35	5 (7.5)	12	35.5 (13.75)	17	13
Custom	12 (12)	50	7.5 (6.75)	38	39.5 (18)	25	33
Cystinosis	2 (2)	50	9 (3)	100	−	−	100
Nephrotic	106 (106)	46	6 (10)	31	41.5 (21)	32	31
NPHP & related ciliopathies	68 (66)	46	6 (8.75)	30	36.5 (14.5)	50	33
Tubulopathies	69 (68)	58	5 (9)	50	31 (21.75)	36	44

A family may be counted in both pediatric and adult cohorts if they have pediatric and adult probands. Diagnostic rate was defined as the proportion of referred families in which a pathogenic or likely pathogenic variant was detected, according to 2015 ACMG guidelines^20^. Sex ratio was abbreviated as %F (percent female). Interquartile range was abbreviated as IQR.

Abbreviated panels are as follows: *ADTKD* autosomal dominant tubulointerstitial kidney disease, *aHUS/C3 GN* atypical hemolytic uremic syndrome-C3 glomerulonephritis, *Alport* Alport syndrome, *ARPKD* autosomal recessive polycystic kidney disease, *BORS* branchio-oto renal syndrome, *CAKUT* congenital anomalies of the kidney and urinary tract, *Nephrotic* nephrotic syndrome, *NPHP & related ciliopathies* nephronophthisis & related ciliopathies.

### Panel data

**Fig. 4:**
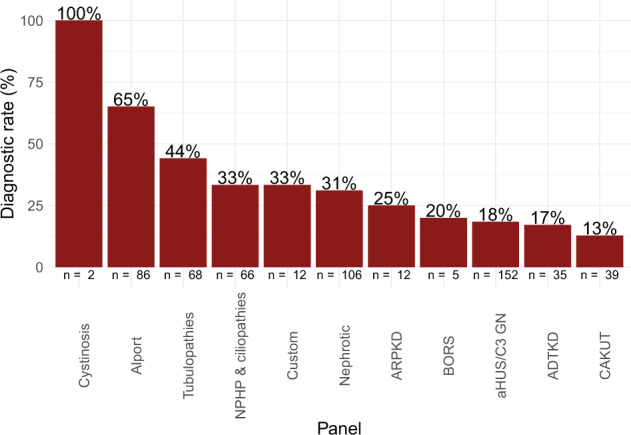
Diagnostic rate of pathogenic or likely pathogenic variants, stratified by panel. Variant classification was based on 2015 ACMG guidelines^20^. The number of families referred for each panel is shown along the *x*-axis. Abbreviated panels are as follows: ADTKD autosomal dominant tubulointerstitial kidney disease, aHUS/C3 GN atypical hemolytic uremic syndrome-C3 glomerulonephritis, Alport Alport syndrome, ARPKD autosomal recessive polycystic kidney disease, BORS branchio-oto renal syndrome, CAKUT congenital anomalies of the kidney and urinary tract, Nephrotic nephrotic syndrome, NPHP & ciliopathies nephronophthisis & related ciliopathies.

594 panels were completed – 92.9% of referrals (513/552) requested a single panel, 6.7% (37/552) requested two panels, and 0.4% (2/552) requested 3 or more panels. Diagnostic rate was defined as the proportion of referred families in which a pathogenic or likely pathogenic variant was detected. Requested panels included atypical hemolytic uremic syndrome-C3 glomerulonephritis (aHUS/C3 GN) (*n* = 154), nephrotic syndrome (NS) (*n* = 106), Alport syndrome (*n* = 90), tubulopathies (*n* = 69), nephronophthisis & related ciliopathies (NPHP-RD) (*n* = 68), congenital anomalies of the kidney and urinary tract (CAKUT) (*n* = 40), autosomal dominant tubulointerstitial kidney disease (ADTKD) (*n* = 36), autosomal recessive polycystic kidney disease (ARPKD) (*n* = 12), custom (*n* = 12), branchio-oto renal syndrome (BORS) (*n* = 5), and cystinosis (*n* = 2). For 12 patients a custom or individualized panel was designed with (and at the discretion of) the referring clinician due to either a lack of clear phenotype (e.g., up to the entire renal gene panel for patients presenting in end stage kidney failure), the presence of two possibly distinct phenotypes (e.g., nephrotic syndrome and congenital cataracts), or where a panel had not yet been designed (e.g., hypomagnesemia). Panels with the highest diagnostic yields for pathogenic or likely pathogenic variants were those for cystinosis (100%, 2/2), Alport syndrome (65%, 56/86), and tubulopathies (44%, 30/68) (Fig. 4, Table 1). aHUS/C3 GN (18%, 28/152), ADTKD (17%, 6/35), and CAKUT (13%, 5/39) recorded the lowest diagnostic rates for pathogenic or likely pathogenic variants across the range of panels offered in our service (Fig. 4, Table 1). Of the detected variants of uncertain significance (VOUS) and (likely) pathogenic variants, variants were most commonly found in *COL4A5* (54/406) and *SLC12A3* (29/406) (Tables 2 and 3). Prior to introduction of whole exome sequencing in April 2018, the diagnostic workflow utilized the TruSight One panel, which captured 4,813 genes that were reported to be associated with Mendelian disorders at the time of manufacture (Illumina Inc., San Diego, CA). This was used as the ‘backbone’ for the sequencing and then appropriate genes were analyzed for each panel. From April 2018 onwards, whole exome sequencing was performed as the ‘backbone’ and analysis has remained targeted to the panels of interest. This enabled an additional 35 genes to be incorporated into existing panels and are highlighted in Supplementary Table 1. The expanded panels on the exome ‘backbone’ have allowed for detection of 2 pathogenic or likely pathogenic variants (aHUS/C3 GN = 1, NS = 1) and 6 VOUS (NPHP-RD = 2, NS = 2, CAKUT = 1, custom = 1) in genes that were previously not included (Methods, Supplementary Table 1).

**Table 2:** Identified pathogenic or likely pathogenic variants stratified by panel.

Panel	%F	Number of families	Overall diagnostic rate (%)	Identified genes (number of families)
ADTKD	67	6	17	*UMOD* (5), *HNF1B* (1)
aHUS/C3 GN	62	28^a^	18	*CFH* (8), *CFHR1* and *CFHR3* homozygous deletion (8), *THBD* (5), *CD46* (3), *C3* (2), *CFHR1* homozygous deletion (1), *CFHR5* (1), *DGKE* (1)
Alport	55	56	65	*COL4A5* (41), *COL4A4* (8), *COL4A3* (7)
ARPKD	67	3	25	*PKHD1* (3)
BORS	0	1	20	*EYA1* (1)
CAKUT	40	5	13	*FRAS1* (1), *GATA3* (1), *HNF1B* (1), partial *HPSE2* homozygous triplication (1)^b^, *PAX2* (1)
Custom	50	4	33	*CASR* (1), *OCRL* (1), *RMND*1 (1), *RUNX2* (1)
Cystinosis	50	2	100	*CTNS* (2)
Nephrotic	42	33	31	*NPHS1* (7), *NPHS2* (5), *COL4A4* (3), *TRPC6* (3), *CLCN5* (2), *COL4A3* (2), *COL4A5* (2), *LAMB2* (2), *COQ8B* (1), *LMX1B* (1), *MYO1*E (1), *PAX2* (1), *PLCE1* (1), *SMARCAL1* (1), *TTC21B* (1)
NPHP & related ciliopathies	41	22	33	*BBS1* (3), *NPHP1* (3), *NPHP4* (3), *BBS10* (2), *AHI1* (1), *ALMS*1 (1), *BBS7* (1), *C5ORF42* (1), *CEP290* (1), *IFT140* (1), *INVS* (1), *IQCB1* (1), *MKKS* (1), *NPHP3* (1), *TMEM67* (1)
Tubulopathies	60	30	44	*SLC12A3* (13), *CLCN5* (3), *AGXT* (2), *HNF4A* (2), *SLC4A1* (2), *SLC7A9* (2), *AQP2* (1), *ATP6V1B1* (1), *CLCNKB* (1), *GRHPR* (1), *SCNN1A* (1), *SLC12A1* (1)

Variant classification was based on 2015 ACMG guidelines^20^. Diagnostic rate was defined as the proportion of referred families in which a pathogenic or likely pathogenic variant was detected. Sex ratio was abbreviated as %F (percent female).

Abbreviated panels are as follows: *ADTKD* autosomal dominant tubulointerstitial kidney disease, *aHUS/C3 GN* atypical hemolytic uremic syndrome-C3 glomerulonephritis, *Alport* Alport syndrome, *ARPKD* autosomal recessive polycystic kidney disease, *BORS* branchio-oto renal syndrome, *CAKUT* congenital anomalies of the kidney and urinary tract, *Nephrotic* nephrotic syndrome, *NPHP & related ciliopathies* nephronophthisis & related ciliopathies.

^a^Two likely pathogenic/pathogenic variants were found in one family.

^b^Unaffected parents confirmed heterozygous, affected siblings also homozygous.

**Table 3:** Identified VOUS stratified by panel.

Panel	%F	Number of families	VOUS identification rate (%)	Identified genes (number of families)
ADTKD	40	5	14	*UMOD* (4), *REN* (1)
aHUS/C3 GN	65	23	15	*CFH* (6), *ADAMTS13* (3), *C3* (3), *CFI* (3), *CD46* (2), *THBD* (2), *CFB* (1), *CFHR1* and *CFHR3* heterozygous deletion (1), *CFHR5* (1), *PLG* (1)
Alport	40	5	6	*COL4A5* (4), *COL4A4* (1)
ARPKD	50	2	17	*PKHD1* (2)
BORS	–	0	0	–
CAKUT	25	16^a^	41	*FRAS1* (3), *ROBO2* (2), *SALL1* (2), *ACE* (1), *BICC1* (1), *FREM1* (1), *FREM2* (1), *HNF1B* (1), *JAG1* (1), *KMT2D* (1), *NIPBL* (1), *NOTCH2* (1), *RET* (1), *SRGAP1* (1)
Custom	0	2	17	*COQ8B* (1), *SEC63*(1)
Cystinosis	–	0	0	*–*
Nephrotic	52	23^b^	22	*TRPC6* (3), *TTC21B* (3), *COL4A5* (2), *INF2* (2), *LAMB2* (2), *NPHS2* (2), *ACTN4* (1), *ALMS*1 (1), *ANLN* (1), *ARHGAP24* (1), *ITGB4* (1), *MYH9* (1), *NPHS1* (1), *NUP93* (1), *PLCE1* (1), *WT1* (1)
NPHP & related ciliopathies	25	142	21	*NPHP4* (5), *C5ORF42* (2), *CC2D2A* (1), *CEP164* (1), *DYNC2H1* (1), *EVC* (1), *IFT172* (1), *INVS* (1), *KIF7* (1), *WDR19* (1)
Tubulopathies	62	13	19	*SLC12A3* (5), *SLC34A1* (2), *SLC4A1* (2), *ATP6V1B1* (1), *CASR* (1), *SLC12A1* (1), *SLC7A9* (1)

Variant classification was based on 2015 ACMG guidelines^20^. Variant of uncertain significance (VOUS) identification rate was defined as the proportion of referred families in which at least one VOUS was detected. Sex ratio was abbreviated as %F (percent female).

Abbreviated panels are as follows: *ADTKD* autosomal dominant tubulointerstitial kidney disease, *aHUS/C3 GN* atypical hemolytic uremic syndrome-C3 glomerulonephritis, *Alport* Alport syndrome, *ARPKD* autosomal recessive polycystic kidney disease, *BORS* branchio-oto renal syndrome, *CAKUT* congenital anomalies of the kidney and urinary tract, *Nephrotic* nephrotic syndrome, *NPHP & related ciliopathies* nephronophthisis & related ciliopathies.

^a^VOUS were identified in two different genes in two families.

^b^VOUS were identified in two different genes in one family.

**Fig. 5:**
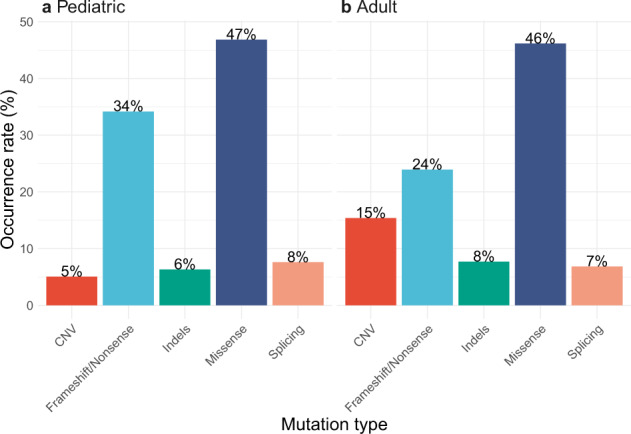
Mutation types of pathogenic or likely pathogenic variants. **a** Distribution of mutation types of pathogenic or likely pathogenic variants in the pediatric cohort (*n* = 281 probands). **b** Distribution of mutation types of pathogenic or likely pathogenic variants in the adult cohort (*n* = 271 probands). Variant classification was based on 2015 ACMG guidelines^20^. Abbreviated mutation types are as follows: CNV, copy number variation, indels insertions or deletions.

The overall genetic diagnostic rate of pathogenic or likely pathogenic variants was 35% (189/542); the pediatric diagnostic rate (38%) was slightly higher than that of adults (32%, *P* = 0.07, Table 1). The diagnostic rate of VOUS was 17% in children and 12% in adults (*P* = 0.05, Table 3). In the event of a VOUS finding, family segregation testing was performed to determine whether the variant was de novo or segregating with the phenotype in the family, if there was a positive family history. In some cases, these have resulted in the reclassification of a variant from VOUS to likely pathogenic or likely benign. Variant reclassification would also be performed upon request from the referring clinician. The percentage of families in which a (likely) pathogenic variant or VOUS was not identified was 45% in the pediatric cohort and 55% in the adult cohort (*P* < 0.002, Table 4). Of the 275 diagnosed pathogenic or likely pathogenic variants, 128 were missense, 82 were frameshift or nonsense, 26 were copy number variations (CNVs), 20 were canonical splice-site variations, and 19 were insertions or deletions (indels); the distribution of mutation types was similar when stratified by age (pediatric vs. adult) (Fig. 5). By comparison, 82% of VOUS were missense (Supplementary Fig. 1). VOUS were identified in over 20 different genes including previously well described genes (e.g., *COL4A5* and *CFH*) (Table 3). Classification of novel missense variants is challenging using the ACMG guidelines^20^, without performing functional studies or wide segregation, particularly for autosomal dominant disease. In addition, ethnicity can challenge variant classification as currently available population datasets are biased to European or Caucasian populations. Where possible, familial segregation was performed to assist in variant interpretation.

**Table 4:** Summary of results in which no (likely) pathogenic variant or VOUS was identified.

Panel	%F	Number of families	None identified (%)
ADTKD	52	24	69
aHUS/C3 GN	63	104	68
Alport	64	28	33
ARPKD	44	9	75
BORS	75	4	80
CAKUT	43	21	54
Custom	67	6	50
Cystinosis	–	0	0
Nephrotic	48	52	49
NPHP & related ciliopathies	56	34	52
Tubulopathies	53	32	47

Variants were classified according to 2015 ACMG guidelines^20^. Sex ratio was abbreviated as %F (percent female). Percentage of families in which no (likely) pathogenic variant or variant of uncertain significance (VOUS) was identified was abbreviated as none identified.

Abbreviated panels are as follows: *ADTKD* autosomal dominant tubulointerstitial kidney disease, *aHUS/C3 GN* atypical hemolytic uremic syndrome-C3 glomerulonephritis, *Alport* Alport syndrome, *ARPKD* autosomal recessive polycystic kidney disease, *BORS* branchio-oto renal syndrome, *CAKUT* congenital anomalies of the kidney and urinary tract, *Nephrotic* nephrotic syndrome, *NPHP & related ciliopathies* nephronophthisis & related ciliopathies.

**Fig. 6:**
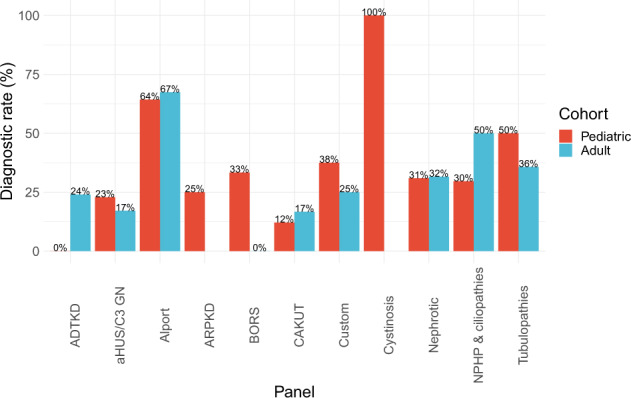
Diagnostic rate of pathogenic or likely pathogenic variants, stratified by panel and cohort. There were 278 families with a pediatric proband and 266 families with an adult proband. Two families had both affected pediatric and adult probands. Variant classification was based on 2015 ACMG guidelines^20^. Abbreviated panels are as follows: ADTKD autosomal dominant tubulointerstitial kidney disease, aHUS/C3 GN atypical hemolytic uremic syndrome-C3 glomerulonephritis, Alport Alport syndrome, ARPKD autosomal recessive polycystic kidney disease, BORS branchio-oto renal syndrome, CAKUT congenital anomalies of the kidney and urinary tract, Nephrotic nephrotic syndrome, NPHP & ciliopathies nephronophthisis & related ciliopathies.

Age-specific diagnostic rates varied by panel (Fig. 6, Table 1). The adult diagnostic rate for nephronophthisis and related ciliopathies was greater than that of children (50 and 30% respectively, Fig. 6, Table 1); conversely, the pediatric diagnostic rate for tubulopathies was greater than that of adults (50 and 36% respectively, Fig. 6, Table 1). Meanwhile, the adult and pediatric diagnostic rates for nephrotic syndrome were similar (32 and 31% respectively, Fig. 6, Table 1).

## Discussion

Here, we determine the utility of targeted exome sequencing by evaluating the results of panel testing conducted by the ANZRGP service on 552 individuals from 542 unrelated families with suspected genetic kidney disease. Our results indicate an overall genetic diagnostic rate of 35% for Australian and New Zealand families referred by their medical practitioner. The pediatric diagnostic rate was 38% and that of adults was 32%. Our diagnostic yield may even underestimate the utility of genetic screening in kidney disease as patients with positive Sanger sequencing or chromosome microarray (CMA) encompassing both array comparative genomic hybridization and single nucleotide polymorphism array results were not subsequently referred for panel testing. In addition, *PKD1* and *PKD2* sequencing are not currently offered using this exome-based pipeline due to pseudogene homology with the *PKD1* gene, which accounts for ~80% of autosomal dominant polycystic kidney disease (ADPKD) disease-causing variants^22^. In addition, high GC-content in both *PKD1* and *PKD2* impacts sequence quality^22^. Our results were comparable to a previous cohort study in Ireland^4^ in which a genetic diagnosis was determined in 42 of 114 (37%) adult families with suspected CKD by whole exome sequencing (WES) and the recent study in China^3^ in which a genetic diagnostic rate of 42.1% was determined in 1001 pediatric patients with suspected genetic kidney disease by a combination of target gene sequence, WES, and trio-WES. These rates were higher than the genetic diagnostic rate of 9.3% determined in 3315 relatively unselected American patients^2^ with CKD and end stage kidney disease, where the lower rate may reflect the greater proportion of CKD secondary to type 2 diabetes and hypertension. Results from the four cohorts indicate that greater selection for sequencing in specialized pediatric and adult kidney genetic clinics and the addition of segregation testing in parents may push diagnostic rates even higher.

The NS panel produced a similar diagnostic yield to that of the Chinese cohort^3^ (31% and 29.1% respectively). Our NS diagnostic rate likely underestimates the diagnostic utility of genetic testing for this condition as most prevalent pediatric patients had been tested using Sanger sequencing of *NPHS1*, *NPHS2*, and/or *WT1* offered by our laboratory and others prior to the introduction of panel testing. Nevertheless, pathogenic or likely pathogenic variants were detected most commonly in *NPHS1* and *NPHS2* while the majority of the variants identified in the Chinese cohort^3^ were found in *COQ8B*, *WT1*, and *NPHS1*, illustrating the utility of panels that cater to patients of different ethnic backgrounds. Referrals for the aHUS/C3 GN panel for adult patients were more than three times that for pediatric patients, reflecting the higher incidence of TMA in adults as well as the need for a genetic diagnosis before Eculizumab^23^ may be prescribed long-term under the Pharmaceutical Benefits Scheme (PBS)^24^ in Australia; this PBS policy results in close to ubiquitous testing in the context of TMA in adults where thrombotic thrombocytopenic purpura has been ruled out^24^. Our aHUS/C3 GN panel reported a diagnostic rate of 18%, higher than a prior report of 11.9%^7^. For nephronophthisis and related ciliopathies our diagnostic rate was 33%, which is comparable with prior reports of 12–25%^14,25–27^. Unexpectedly, the rate was higher in adult patients referred. This is likely due in part to selection bias (only 14 adult patients were referred compared with 54 pediatric patients). However, it is also increasingly recognized that the first presentation of nephronophthisis may be in adulthood particularly when non-syndromic and thus referrals are likely to increase in number^28,29^. The number of females being referred for Alport gene panel testing is at odds with the male preponderance of the condition due to the frequency of X-linked (*COL4A5*) disease. However, as demonstrated in Fig. 3, the age at referral differs between males and females. This likely represents adult females with microscopic hematuria being referred because of the benefit from a gene test over a more invasive renal biopsy in terms of informing not only their own risk of future disease progression but risk to relatives including for the purpose of family planning. Our CAKUT panel processed 39 families in comparison to disorders with clearer clinical phenotypes such as Alport syndrome (*n* = 86) and NS (*n* = 106). The CAKUT panel produced a diagnostic yield of 13%, comparable to 17% in a prior cohort study^3^. As a negative CMA result was required prior to panel testing to rule out recurrent variants such as 17q12 or 7q11.23 deletions, our diagnostic rate likely underestimates the utility of the CAKUT panel. Exome sequencing for monogenic disease-causing variants has not proved as insightful for CAKUT as for all other renal disorders. This is despite the known association with recurrent copy number variants, the frequency of bilateralism, and a positive family history in up to 25% of patients^30^. Variable penetrance and variable phenotype within families increase the complexity but it is likely that epigenetic and environmental factors play a significant role which is yet to be delineated^31,32^. Future testing that accounts for polygenic relationships may also increase the utility of genetic testing.

Furthermore, diagnostic rates for Alport syndrome and ARPKD were lower than those of prior reports^33,34^; however, for the Alport panel this may be attributed to patients with suspected thin basement membrane nephropathy being referred for testing prior to histological confirmation; while for the ARPKD panel, the small cohort size (*n* = 12) prevents clear interpretation. In addition, some of the variants that were classified as VOUS may be re-classified in the future as novel pathogenic or likely pathogenic variants as a result of functional analysis or international cohort analysis^35^. For tubulopathies, our diagnostic rate of 44% was comparable to that of a previous cohort study^3^. The cohort size (*n* = 2) of the cystinosis panel is not large enough to make reasonable comparisons. For ADTKD our diagnostic yield was lower than a previous report^10^, likely due to the inability to sequence *MUC1*, a major causal gene, using an exome approach as well as prior detection by CMA of deletions and the availability of Sanger sequencing of *HNF1B*.

Our results indicate that targeted exome sequencing is useful for diagnosing kidney disorders at the clinical level. Increasingly, panel results may help inform appropriate treatment. For patients with nephrotic syndrome, not identifying a genetic etiology is key. However, new genes continue to be described frequently, thus the panel requires regular update and reanalysis of the new genes may be appropriate for patients when genetic disease is considered likely clinically and nothing had been identified on previous sequencing. Despite this, if no variant is identified it may suggest an immunological or as yet unidentified cause and supports the ongoing use of immunosuppressive therapies, but also indicates a high risk of recurrence post-transplant; while a genetic cause would advise the contrary. Testing results may also further encourage reverse phenotyping^36^, or the subsequent review of extra-renal features; for instance, a pathogenic variant detected in *HNF1B* would prompt monitoring for diabetes^37^ while a diagnosis of a ciliopathy such as Bardet-Biedl syndrome (BBS) would prompt additional consultations (e.g., ophthalmic)^38^. Furthermore, by targeting a curated list of genes, panels may be administered at a controlled cost of AUD$1500-$2000 and shorten the time required to diagnose the etiology of the disorder. This targeted approach also reduces the risk of incidental or secondary findings. By identifying the molecular cause of the clinical presentation, the specific subtype of the kidney disorder may also be determined or even be re-classified as a different disease; this precise method of diagnosis may help to inform family planning, support cases for wider cascade testing, and predict future disease progression. In planning for kidney transplantation, a molecular diagnosis may inform risk of disease recurrence. Additionally, if the mutation disrupts the protein severely enough, a transplant will expose the recipient to de novo antigens from the donor kidney, such as has been reported with *NPHS1* (congenital nephrotic syndrome) and *COL4A5* (Alport syndrome)^39–43^. Although gene therapy for renal disorders does not currently exist, the expansion in this field of research and the success seen in other specialties suggest it will become feasible in the future^44^. A molecular diagnosis will then be essential to stratify patients. Targeted sequencing may also function as an alternative to invasive diagnostic procedures such as obtaining biopsies particularly in late presentation end stage kidneys when biopsies are typically less informative. The utility of this service will be further extended by the planned introduction of panels for ADPKD and renovascular disease.

However, there are various clinical, funding, and analysis issues that limit gene testing for kidney disease currently. Variability in state health funding policies may influence grounds for referral^45^. As patients were referred at their clinician’s discretion, there may be referral biases in which certain clinical symptoms are overrepresented in panel testing data. Some patients may have been referred for testing despite a lack of evidence for the clinical phenotype, which reduces the diagnostic yield of the tests. Additionally, some regions are difficult to sequence. For example, homologous regions in *CFH*, *CFHR1*, and *CFHR3* require additional Sanger sequencing to verify the results^46^. Due to pseudogene homology^47^, *PKD1* sequencing is unreliable using the current methodology described. However, for introduction into this clinical service, whole genome sequencing techniques^22^ have been developed to overcome this issue. Furthermore, sequencing errors of structural rearrangements and copy number variations may be better detected with improved alignment algorithms as well as orthogonal methods such as multiplex ligation-dependent probe amplification (MLPA) and ultra-high resolution arrays; for example, MLPA may be used to detect *CFHR* gene rearrangements^48^ in the aHUS/C3 GN panel. Further studies should also consider the utility of intronic sequencing and whole genome sequencing in diagnosing the molecular basis of the condition in cases where no variant has been identified by existing methods. Furthermore, due to the lack of available supporting literature or functional analysis, some novel missense variants may have been interpreted as VOUS. This limitation is further compounded by the ethnically diverse population of Australia and New Zealand; classifying novel missense variants in patients with ethnic backgrounds underrepresented in population databases^49^ remains a challenge even with the use of segregation analysis. Hence, there is a need for not only more genetic research literature to aid variant interpretation but also increased inclusion of previously underrepresented populations in genetic research and reference databases. Finally, an emphasis on documenting a priori clinical diagnoses and phenotypic presentations in future studies would allow for richer analysis of the data.

The current methodology allows for extended analysis where clinical phenotype is difficult to define such that analysis of all genes associated with kidney disease is possible if required. Similarly, reanalysis is possible if a chosen panel returns a negative result or the clinical phenotype changes with time.

Evidence of the rapid uptake of sequencing as a diagnostic tool in routine clinical practice is provided by the number of individual referrers. The results suggest pediatric nephrologists may be more comfortable referring for sequencing as they referred 5 more patients on average than adult nephrologists. However, this likely reflects a single time point in a changing environment where adult nephrologists both in and out of the public health system feel increasingly comfortable ordering these tests. Historically, nephrology training both in Australasia and abroad has not considered genomics a core unit yet in the authors’ opinion a genetic test cannot be considered equivalent to other kidney investigations given the complexity and potential ramifications for a proband and their extended family. A number of educational tools have become available within Australia, developed by the Royal Australian College of Physicians and Australian Genomics, which provide an introduction to key concepts in genomics for health professionals. In addition, future work by the KidGen collaborative will explore information requirements and development of an educational framework for nephrology trainees.

In summary, results from targeted exome sequencing of over 230 kidney genes in 552 individuals from 542 unrelated families with suspected kidney disease in Australia and New Zealand demonstrate the utility of panel testing for these disorders. Relatively, the CAKUT panel has less utility given the poor yield beyond recurrent copy number variation, such that identifiable (likely) pathogenic variants remain rare despite an increasingly comprehensive gene panel. We report an overall diagnostic rate of 35% in adult and pediatric probands by the ANZRGP service, a diagnostic genetic testing service with clinical accreditation, using data from the largest cohort of Australian and New Zealand kidney patients reported to date. Primarily referrals for testing came from nephrologists both independent and within a kidney genetic clinic. The results were comparable to previous international cohort studies^2–4^. Access to high throughput genetic testing has been revolutionary for families with kidney disease and their treating teams. While in most cases the results are primarily diagnostic, increasingly they will play an important part in directing treatment, stratifying patients for and within therapeutic trials, avoiding inappropriate or futile treatments, and allowing ongoing genetic counseling and family planning.

## Methods

### Referrals

Referrals received between December 2013 and October 2019 were processed at the Molecular Genetics Laboratory of the Children’s Hospital at Westmead (Sydney, NSW, Australia). Referral requirements were as previously described^5^, including the isolated DNA sample of the patient from the referring institution, details of the panel requested, working diagnosis, relevant clinical data, and completed clinical genetic testing consent forms. Referral was open to all doctors throughout Australia and New Zealand and advertised to nephrologists and geneticists. All referrals were reviewed to confirm minimum information provision and suitability. Panel results were returned to the referring clinician complete with variant interpretation. Patient data was reanalyzed upon request. Segregation testing of any variants identified in other family members was carried out using Sanger sequencing as per standard laboratory protocols.

### Multigene panel curation

Gene lists for each panel were curated according to the ClinGen criteria^50^ by a multidisciplinary team of nephrologists, clinical geneticists, and laboratory scientists. Known disease-causing variants, functional studies, and segregation analyses were considered prior to the inclusion of any particular gene in the panel. Gene lists are regularly reviewed and updates may include the addition or removal of genes from the list.

### Genetic sequencing

Between December 2013 and April 2018, the diagnostic service utilized the Nextera Capture method of Illumina TruSight One and Illumina TruSight Clinical Exomes as previously described^5^. This service was designed to focus on disease-causing genes, including those in Human gene mutation database (http://www.hgmd.org/) and OMIM (http://www.omim.org/). The TruSight Clinical Exome captured 2,761 OMIM-identified disease genes and the expanded TruSight One targeted 4,813 disease genes. All clinical exome samples were sequenced using the Illumina HiSeq 2500 (Illumina Inc.) or the Illumina NextSeq500 (Illumina Inc.) with a 2 × 150-base paired–end read protocol (Ramaciotti Centre, Sydney, NSW, Australia). Since April 2018, library preparation and capture for whole exome sequencing have been performed at the Kinghorn Centre of Clinical Genomics at the Garvan Institute of Medical Research (Sydney, NSW, Australia) using Agilent SureSelect Clinical Research Exome (v2). Sequencing was performed on Illumina HiSeqX to an average depth of coverage of 100x across captured regions. Alignment and variant calling were performed using SoftGenetics NextGene, using the February 2009 human genome assembly (GRCh37/hg19) and the latest version of the software at time of testing (up to v2.4.1). Variant interpretation was restricted to coding regions and the canonical splice sites of panel of genes. Variant genotyping was supplemented with gap filling for certain genes. Copy number variation (CNV) analyses were also performed using the ‘Batch CNV Tool’ within the NextGene software, which was specific for copy number variants affecting 3 or more consecutive exons, although single exon CNVs had also been detected as part of other routine laboratory testing. All putative CNVs were investigated using an orthogonal method, either multiplex ligation-dependent probe amplification (MLPA) or CMA as per standard laboratory protocols, prior to reporting. Alignment and variant calling were performed using the most recent version of the software at time of testing.

### Variant filtering & analyses

To minimize variant classification errors due to poor read quality or sequencing errors, variants with less than 15x depth of coverage were removed from analysis. Automated variant annotation including minor allele frequency was performed using Alamut Batch (inputting from the SNP database^51^, 1000 Genomes^52^, Exome Variant Server^53^, Exome Aggregation Consortium^54^ and the Genome Aggregation Database^55^). Heterozygous variants associated with dominant and recessive disorders were filtered out based on mean allele frequency (>0.05% and >1% respectively) and pathogenicity classification on ClinVar^18^.

Pathogenicity was assessed with the help of Alamut Visual, taking into account literature, computational, functional, and population data. Variants were classified according to 2015 ACMG guidelines^20^. If a variant was found to be pathogenic or likely pathogenic, Sanger sequencing was employed to confirm the variant. All analyses were performed using the most recent version of the software at time of testing.

### Statistical analyses

Statistical analyses and data visualizations were performed using R (v3.6.1; https://www.R-project.org). Comparisons were performed using the chi-square test for categorical variables (one-sided). Diagnostic rate was defined as the proportion of referred families in which a pathogenic or likely pathogenic variant was detected according to 2015 ACMG guidelines for clinical interpretation^20^. Percentages were rounded to the nearest percent unless otherwise stated.

### Ethical approval

All participants were consented for genetic testing by their local team prior to referral.

This study was approved by the Sydney Children’s Hospitals Network (SCHN) Human Research Ethics Committee (LNR/15/SCHN/505) and Research Governance Committee (LNFSSA/16/SCHN/62) and included a waiver of consent for publication of the variants due to the de-identified nature of the data.

### Reporting summary

Further information on research design is available in the Nature Research Reporting Summary linked to this article.

## Supplementary information

Supplementary Information

Reporting Summary

## Data Availability

The datasets generated and/or analyzed during the current study have been uploaded to www.shariant.org.au (the Australian Genomics Variant Classification Sharing Platform) and deposited into ClinVar (https://www.ncbi.nlm.nih.gov/clinvar/) (accession numbers SCV001449172–SCV001449477). They are also available from the corresponding author on reasonable request.

## References

[1] Chen W (2009). Prevalence and risk factors associated with chronic kidney disease in an adult population from southern China. Nephrol. Dial. Transplant..

[2] Groopman EE (2019). Diagnostic utility of exome sequencing for kidney disease. N. Engl. J. Med..

[3] Rao J (2019). Genetic spectrum of renal disease for 1001 Chinese children based on a multicenter registration system. Clin. Genet..

[4] Connaughton DM (2019). Monogenic causes of chronic kidney disease in adults. Kidney Int..

[5] Mallett AJ (2017). Massively parallel sequencing and targeted exomes in familial kidney disease can diagnose underlying genetic disorders. Kidney Int..

[6] Braun DA (2016). Prevalence of monogenic causes in pediatric patients with nephrolithiasis or nephrocalcinosis. Clin. J. Am. Soc. Nephrol..

[7] Bu F (2016). High-throughput genetic testing for thrombotic microangiopathies and C3 glomerulopathies. J. Am. Soc. Nephrol..

[8] Bu F (2014). Comprehensive genetic analysis of complement and coagulation genes in atypical hemolytic uremic syndrome. J. Am. Soc. Nephrol..

[9] Ece Solmaz A (2015). Targeted multi-gene panel testing for the diagnosis of Bardet Biedl syndrome: Identification of nine novel mutations across BBS1, BBS2, BBS4, BBS7, BBS9, BBS10 genes. Eur. J. Med. Genet..

[10] Ekici AB (2014). Renal fibrosis is the common feature of autosomal dominant tubulointerstitial kidney diseases caused by mutations in mucin 1 or uromodulin. Kidney Int..

[11] Klingbeil KD (2017). Novel EYA1 variants causing Branchio-oto-renal syndrome. Int J. Pediatr. Otorhinolaryngol..

[12] Mencarelli MA (2015). Evidence of digenic inheritance in Alport syndrome. J. Med. Genet..

[13] Sadowski CE (2015). A single-gene cause in 29.5% of cases of steroid-resistant nephrotic syndrome. J. Am. Soc. Nephrol..

[14] Schueler M (2016). Large-scale targeted sequencing comparison highlights extreme genetic heterogeneity in nephronophthisis-related ciliopathies. J. Med. Genet..

[15] van der Ven AT, Vivante A, Hildebrandt F (2018). Novel insights into the pathogenesis of monogenic congenital anomalies of the kidney and urinary tract. J. Am. Soc. Nephrol..

[16] Harris PC, Rossetti S (2010). Molecular diagnostics for autosomal dominant polycystic kidney disease. Nat. Rev. Nephrol..

[17] Ashton EJ (2018). Simultaneous sequencing of 37 genes identified causative mutations in the majority of children with renal tubulopathies. Kidney Int..

[18] Landrum MJ (2014). ClinVar: public archive of relationships among sequence variation and human phenotype. Nucleic Acids Res..

[19] Hamosh A, Scott AF, Amberger JS, Bocchini CA, McKusick VA (2005). Online Mendelian Inheritance in Man (OMIM), a knowledgebase of human genes and genetic disorders. Nucleic Acids Res..

[20] Richards S (2015). Standards and guidelines for the interpretation of sequence variants: a joint consensus recommendation of the American College of Medical Genetics and Genomics and the Association for Molecular Pathology. Genet. Med..

[21] Bruel A (2017). Hemolytic uremic syndrome in pregnancy and postpartum. Clin. J. Am. Soc. Nephrol..

[22] Mallawaarachchi AC (2016). Whole-genome sequencing overcomes pseudogene homology to diagnose autosomal dominant polycystic kidney disease. Eur. J. Hum. Genet..

[23] Greenbaum LA (2016). Eculizumab is a safe and effective treatment in pediatric patients with atypical hemolytic uremic syndrome. Kidney Int..

[24] Fox LC (2018). Consensus opinion on diagnosis and management of thrombotic microangiopathy in Australia and New Zealand. Intern. Med. J..

[25] Halbritter J (2012). High-throughput mutation analysis in patients with a nephronophthisis-associated ciliopathy applying multiplexed barcoded array-based PCR amplification and next-generation sequencing. J. Med. Genet..

[26] Halbritter J (2013). Identification of 99 novel mutations in a worldwide cohort of 1,056 patients with a nephronophthisis-related ciliopathy. Hum. Genet..

[27] Otto EA (2011). Mutation analysis of 18 nephronophthisis associated ciliopathy disease genes using a DNA pooling and next generation sequencing strategy. J. Med. Genet..

[28] Hudson R (2020). Adult-diagnosed nonsyndromic nephronophthisis in australian families caused by biallelic NPHP4 variants. Am. J. Kidney Dis..

[29] Snoek R (2018). NPHP1 (Nephrocystin-1) gene deletions cause adult-onset ESRD. J. Am. Soc. Nephrol..

[30] Bulum B (2013). High frequency of kidney and urinary tract anomalies in asymptomatic first-degree relatives of patients with CAKUT. Pediatr. Nephrol..

[31] Capone, V. P., Morello, W., Taroni, F. & Montini, G. Genetics of congenital anomalies of the kidney and urinary tract: the current state of play. Int. J. Mol. Sci. 10.3390/ijms18040796 (2017). 10.3390/ijms18040796 PMC5412380 28398236

[32] Westland, R., Renkema, K. Y. & Knoers, N. Clinical integration of genome diagnostics for congenital anomalies of the kidney and urinary tract. Clin. J. Am. Soc. Nephrol. 10.2215/cjn.14661119 (2020). 10.2215/CJN.14661119 PMC7792653 32312792

[33] Morinière V (2014). Improving mutation screening in familial hematuric nephropathies through next generation sequencing. J. Am. Soc. Nephrol..

[34] Obeidova, L. et al. Molecular genetic analysis of PKHD1 by next-generation sequencing in Czech families with autosomal recessive polycystic kidney disease. BMC Med. Genet. 10.1186/s12881-015-0261-3 (2015). 10.1186/s12881-015-0261-3 PMC4689053 26695994

[35] Smith ED (2017). Classification of genes: standardized clinical validity assessment of gene-disease associations aids diagnostic exome analysis and reclassifications. Hum. Mutat..

[36] de Goede C (2016). Role of reverse phenotyping in interpretation of next generation sequencing data and a review of INPP5E related disorders. Eur. J. Paediatr. Neurol..

[37] Verhave JC, Bech AP, Wetzels JFM, Nijenhuis T (2016). Hepatocyte nuclear factor 1B–associated kidney disease: more than renal cysts and diabetes. J. Am. Soc. Nephrol..

[38] Weihbrecht, K. et al. Keeping an eye on Bardet-Biedl syndrome: A Comprehensive Review of the Role of Bardet-Biedl Syndrome Genes in the Eye. Med. Res. Arch. 10.18103/mra.v5i9.1526 (2017). 10.18103/mra.v5i9.1526 PMC5814251 29457131

[39] Gross O, Weber M, Fries JWU, Müller G-A (2009). Living donor kidney transplantation from relatives with mild urinary abnormalities in Alport syndrome: long-term risk, benefit and outcome. Nephrol. Dial. Transplant..

[40] Browne G (2004). Retransplantation in Alport post-transplant anti-GBM disease. Kidney Int..

[41] Brainwood D, Kashtan C, Gubler MC, Turner AN (1998). Targets of alloantibodies in Alport anti-glomerular basement membrane disease after renal transplantation. Kidney Int..

[42] Pedchenko V (2010). Molecular architecture of the Goodpasture autoantigen in anti-GBM nephritis. N. Engl. J. Med..

[43] Holmberg C, Jalanko H (2014). Congenital nephrotic syndrome and recurrence of proteinuria after renal transplantation. Pediatr. Nephrol..

[44] Karimian A (2019). CRISPR/Cas9 technology as a potent molecular tool for gene therapy. J. Cell. Physiol..

[45] Buchan HA, Duggan A, Hargreaves J, Scott IA, Slawomirski L (2016). Health care variation: time to act. Med. J. Aust..

[46] Díaz-Guillén MA, Rodríguez de Córdoba S, Heine-Suñer D (1999). A radiation hybrid map of complement factor H and factor H-related genes. Immunogenetics.

[47] Ali, H. et al. PKD1 Duplicated regions limit clinical utility of whole exome sequencing for genetic diagnosis of autosomal dominant polycystic kidney disease. Sci. Rep. 10.1038/s41598-019-40761-w (2019). 10.1038/s41598-019-40761-w PMC6412018 30858458

[48] Valoti E (2015). A novel atypical hemolytic uremic syndrome-associated hybrid CFHR1/CFH gene encoding a fusion protein that antagonizes factor H-dependent complement regulation. J. Am. Soc. Nephrol..

[49] Sirugo G, Williams SM, Tishkoff SA (2019). The missing diversity in human genetic studies. Cell.

[50] Strande NT (2017). Evaluating the clinical validity of gene-disease associations: an evidence-based framework developed by the clinical genome resource. Am. J. Hum. Genet..

[51] Sherry ST (2001). dbSNP: the NCBI database of genetic variation. Nucleic Acids Res..

[52] Auton A (2015). A global reference for human genetic variation. Nature.

[53] Fu W (2013). Analysis of 6,515 exomes reveals the recent origin of most human protein-coding variants. Nature.

[54] Lek M (2016). Analysis of protein-coding genetic variation in 60,706 humans. Nature.

[55] Karczewski KJ (2020). The mutational constraint spectrum quantified from variation in 141,456 humans. Nature.

