# Supplementary material for: Australia and New Zealand renal gene panel testing in routine clinical practice of 542 families
Source: NPJ Genom Med. 2021 Mar 4;6:20. doi: 10.1038/s41525-021-00184-x (PMC7933190; doi:10.1038/s41525-021-00184-x)
Supplement: Supplementary file 1 — Supplementary Information [file 41525_2021_184_MOESM1_ESM.pdf]

Supplementary Information

Supplementary Table 1. Gene lists by panel. Genes listed in bold were not included in the TruSight One panel testing prior to April 2018. Abbreviations are as follows: ADTKD, autosomal dominant tubulointerstitial kidney disease; aHUS/C3 GN, atypical hemolytic uremic syndrome-C3 glomerulonephritis; ARPKD, autosomal recessive polycystic kidney disease; BORS, branchio-oto renal syndrome; CAKUT, congenital anomalies of the kidney and urinary tract.

Panel	Gene list
**ADTKD**	*HNF1B, REN,* ***SEC61A1****, UMOD*
**aHUS/C3 GN**	*ADAMTS13, C3, C5_ex21, CD46, CFB, CFH, CFHR1, CFHR2, CFHR3, CFHR5, CFI,* ***DGKE****, MMACHC, THBD* (*PLG* removed)
**Alport syndrome**	*COL4A3, COL4A4, COL4A5*
**ARPKD**	***DZIP1L****, PKHD1*
**BORS**	*EYA1, SIX1, SIX5*
**CAKUT**	*ACE, AGT, AGTR1, BICC1, BMP4, BMP7,* ***CDC5L****, CHD1L, CHD7, DHCR7,* ***DSTYK****, EYA1, EZH2, FAM58A, FGF10, FGF20, FGF8, FGFR1, FGFR2, FOXC1, FOXC2, FRAS1, FREM1, FREM2, GATA3, GLI3, GPC3, GRIP1, HNF1B, HOXA13, HOXA4, HOXB6, HPSE2,* ***ITGA8****, JAG1, KAL1, KDM6A, KMT2D,* ***LRIG2****, LRP4, NIPBL, NOTCH2, NPHP3, PAX2,* ***PBX1****, REN, RET, ROBO2, ROR2, SALL1, SALL4, SEMA3A, SIX1, SIX2, SIX5,* ***SLIT2****, SOX17* ***SRGAP1****, TBC1D1,* ***TBX18****, TFAP2A,* ***TRAP1****, UMOD, UPK3A, WNT4, WT1*
**Cystinosis**	*CTNS*
**Nephronophthisis & related ciliopathies**	*AHI1, ALMS1, ARL13B, ARL6, B9D1, B9D2,* ***BBIP1****, BBS1, BBS10,* ***BBS12****, BBS2, BBS4, BBS5, BBS7,* ***BBS9****, CC2D2A, CCDC28B, CEP164, CEP290, CEP41, CPLANE1, DCDC2, DYNC2H1, GLIS2, IFT122, IFT140,* ***IFT172****,* ***IFT27****, IFT43,* ***IFT74****, INVS, IQCB1, KIF7, LZTFL1,*

	MKKS, MKS1, NEK1, NEK8, NPHP1, NPHP3, NPHP4, RPGRIP1L, SDCCAG8, TCTN1, TCTN2, TMEM138, TMEM216, TMEM237, TMEM67, TTC21B, TTC8, WDPCP, WDR19, WDR35, XPNPEP3 (*EVC* removed)
**Nephrotic syndrome**	*ACTN4, ALG1, ALMS1,* ***ANLN****, APOL1, ARHGAP24,* ***ARHGDIA****, CD2AP, CLCN5, COL4A3, COL4A4, COL4A5, COQ2, COQ6,* ***COQ7****,* ***COQ8B****,* ***CRB2****, CUBN,* ***DGKE****,* ***EMP2****,* ***FAT1****, INF2, ITGB4, KANK1,* ***KANK2****,* ***KANK4****, LAMB2, LMNA, LMX1B, MAGI2, MYH9, MYO1E, NPHS1, NPHS2,* ***NUP107****,* ***NUP205****,* ***NUP93****, OCRL, PAX2, PDSS2, PLCE1, PTPRO, SCARB2, SMARCAL1, TRPC6, TTC21B, WT1,* ***XPO5***
**Tubulopathies**	*ADCY10, AGXT, ALPL, AP2S1, AQP2, APRT, ATP6V0A4, ATP6V1B1, AVPR2, BSND, CA2, CASR, CDC73, CLCN5, CLCNKA, CLCNKB,* ***CLDN10****, CLDN16, CLDN19, CNNM2, CUL3, CYP24A1, CYP27B1, DMP1,* ***EHHADH****, ENPP1, FAH, FAM111A, FAM20A, FGF23, FXYD2,* ***GNA11****, GRHPR, HNF1B, HNF4A, HOGA1, HPRT1, KCNA1, KCNJ1, KCNJ10, KL, KLHL3,* ***MAGED2****, MAGT1, NR3C2, OCRL, PCBD1, PHEX, PTH1R, SCNN1A, SCNN1B, SCNN1G, SLC12A1, SLC12A3, SLC22A12, SLC26A1, SLC2A9, SLC34A1, SLC34A3, SLC3A1, SLC4A1, SLC4A4, SLC7A9, SLC9A3R1, TRPM6, VDR, WNK1, WNK4, XDH*

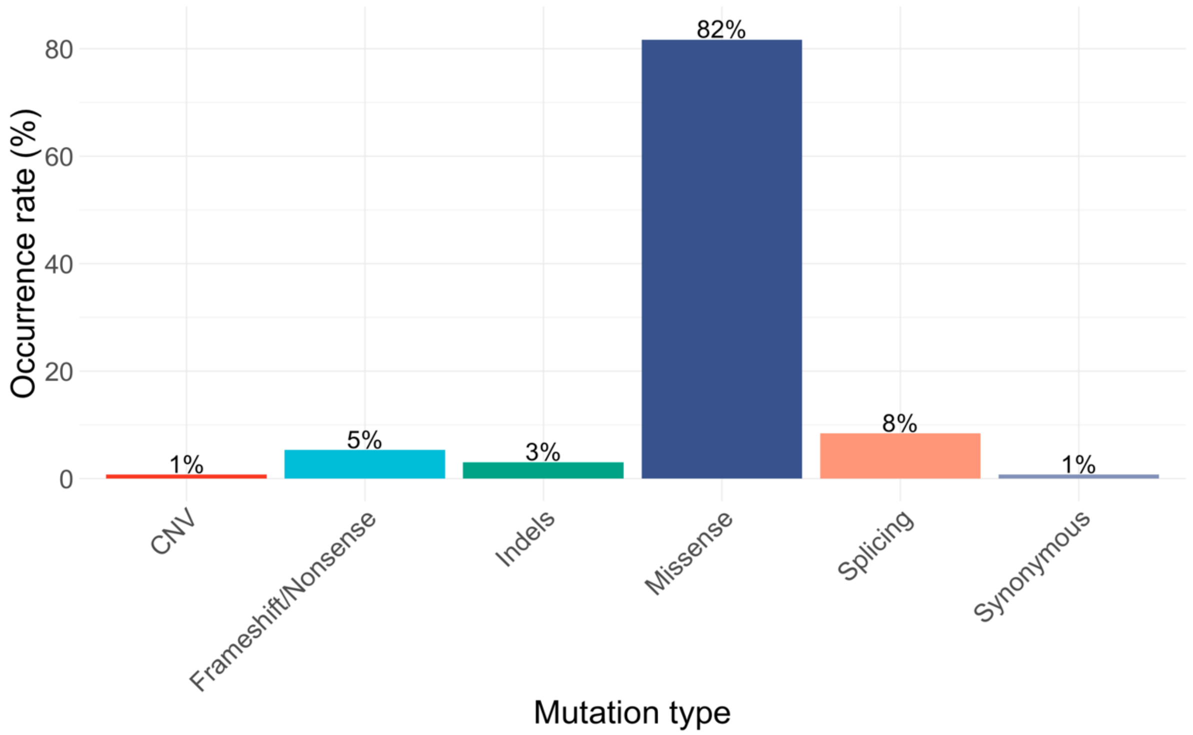

Supplementary Figure 1. Distribution of mutation types of variants of uncertain significance (VOUS) (n = 552 probands). Variant classification was based on 2015 ACMG guidelines[20]. Abbreviated mutation types are as follows: CNV, copy number variation; indels, insertions or deletions.
