# Supplementary material for: Australia and New Zealand renal gene panel testing in routine clinical practice of 542 families
Source: NPJ Genom Med. 2021 Mar 4;6:20. doi: 10.1038/s41525-021-00184-x (PMC7933190; doi:10.1038/s41525-021-00184-x)
Supplement: Supplementary file 2 — Reporting Summary [file 41525_2021_184_MOESM2_ESM.pdf]

nature research

Corresponding author(s): Professor Stephen Alexander and Dr Hugh McCarthy

Last updated by author(s): Aug 13, 2020

Reporting Summary

Nature Research wishes to improve the reproducibility of the work that we publish. This form provides structure for consistency and transparency in reporting. For further information on Nature Research policies, see Authors & Referees and the Editorial Policy Checklist.

Statistics

For all statistical analyses, confirm that the following items are present in the figure legend, table legend, main text, or Methods section.

n/a	Confirmed	
☐	☒	The exact sample size (*n*) for each experimental group/condition, given as a discrete number and unit of measurement
☐	☒	A statement on whether measurements were taken from distinct samples or whether the same sample was measured repeatedly
☐	☒	The statistical test(s) used AND whether they are one- or two-sided<br>*Only common tests should be described solely by name; describe more complex techniques in the Methods section.*
☒	☐	A description of all covariates tested
☒	☐	A description of any assumptions or corrections, such as tests of normality and adjustment for multiple comparisons
☐	☒	A full description of the statistical parameters including central tendency (e.g. means) or other basic estimates (e.g. regression coefficient) AND variation (e.g. standard deviation) or associated estimates of uncertainty (e.g. confidence intervals)
☒	☐	For null hypothesis testing, the test statistic (e.g. *F*, *t*, *r*) with confidence intervals, effect sizes, degrees of freedom and *P* value noted<br>*Give P values as exact values whenever suitable.*
☒	☐	For Bayesian analysis, information on the choice of priors and Markov chain Monte Carlo settings
☒	☐	For hierarchical and complex designs, identification of the appropriate level for tests and full reporting of outcomes
☒	☐	Estimates of effect sizes (e.g. Cohen's *d*, Pearson's *r*), indicating how they were calculated

Our web collection on statistics for biologists contains articles on many of the points above.

Software and code

Policy information about availability of computer code

Data collection	No software used
Data analysis	Calculations and visualizations were performed using R (v3.6.1).

For manuscripts utilizing custom algorithms or software that are central to the research but not yet described in published literature, software must be made available to editors/reviewers. We strongly encourage code deposition in a community repository (e.g. GitHub). See the Nature Research guidelines for submitting code & software for further information.

Data

Policy information about availability of data

All manuscripts must include a data availability statement. This statement should provide the following information, where applicable:

- Accession codes, unique identifiers, or web links for publicly available datasets
- A list of figures that have associated raw data
- A description of any restrictions on data availability

The datasets generated during and/or analysed during the current study have been uploaded to www.shariant.org.au (the Australian Genomics Variant Classification Sharing Platform) and deposited into to ClinVar (https://www.ncbi.nlm.nih.gov/clinvar/) (accession numbers SCV001449172–SCV001449477). They are also available from the corresponding author on reasonable request.

Field-specific reporting

Please select the one below that is the best fit for your research. If you are not sure, read the appropriate sections before making your selection.

☒ Life sciences ☐ Behavioural & social sciences ☐ Ecological, evolutionary & environmental sciences

For a reference copy of the document with all sections, see nature.com/documents/nr-reporting-summary-flat.pdf

Life sciences study design

All studies must disclose on these points even when the disclosure is negative.

Sample size	The study was conducted on 552 individuals from 542 families in Australia and New Zealand.
Data exclusions	Referral requests with incomplete fields such as missing referring doctor were excluded from analysis.
Replication	Analysis was undertaken on the sample of 552 individuals. Due to the nature of the study, no replication was performed.
Randomization	The study was not randomized. Referral was open to doctors from throughout Australia and New Zealand. Referrals with complete information received between December 2013 and October 2019 were included in this study.
Blinding	Blinding is not relevant to the study.

Reporting for specific materials, systems and methods

We require information from authors about some types of materials, experimental systems and methods used in many studies. Here, indicate whether each material, system or method listed is relevant to your study. If you are not sure if a list item applies to your research, read the appropriate section before selecting a response.

Materials & experimental systems

n/a	Involved in the study	
☒	☐	Antibodies
☒	☐	Eukaryotic cell lines
☒	☐	Palaeontology
☒	☐	Animals and other organisms
☐	☒	Human research participants
☐	☒	Clinical data

Methods

n/a	Involved in the study	
☒	☐	ChIP-seq
☒	☐	Flow cytometry
☒	☐	MRI-based neuroimaging

Human research participants

Policy information about studies involving human research participants

Population characteristics	552 individuals (271 adults and 281 pediatric patients) from 542 unrelated families with suspected kidney disease from all Australian states and territories and New Zealand were included in this study.
Recruitment	Patient referral for panel testing was open to doctors from throughout Australia and New Zealand.
Ethics oversight	This study was approved by the Sydney Children's Hospitals Network (SCHN) Human Research Ethics Committee (LNR/15/SCHN/505) and Research Governance Committee (LNFSSA/16/SCHN/62) with waiver of consent for publication due to the de-identified nature of the data.

Note that full information on the approval of the study protocol must also be provided in the manuscript.

Clinical data

Policy information about clinical studies

All manuscripts should comply with the ICMJE guidelines for publication of clinical research and a completed CONSORT checklist must be included with all submissions.

Clinical trial registration	*Provide the trial registration number from ClinicalTrials.gov or an equivalent agency.*
Study protocol	*Note where the full trial protocol can be accessed OR if not available, explain why.*
Data collection	Referrals received between December 2013 and October 2019 were processed at the Molecular Genetics Laboratory of the Children's Hospital at Westmead (Sydney, NSW, Australia). Referral requirements included the isolated DNA sample of the

patient from the referring institution, details of the panel requested, working diagnosis, relevant clinical data, and completed clinical genetic testing consent forms. Referral was open to medical practitioners in Australia and New Zealand.

Outcomes	This was an observational study and as such had no primary or secondary outcomes.
